# *Ridleyandra chuana* (Gesneriaceae), a new species from Peninsular Malaysia

**DOI:** 10.3897/phytokeys.25.5178

**Published:** 2013-07-10

**Authors:** Ruth Kiew

**Affiliations:** 1 Forest Research Institute Malaysia, 52109 Kepong, Selangor, Malaysia

**Keywords:** *Ridleyandra chuana*, Gesneriaceae, Peninsular Malaysia, endemic, conservation

## Abstract

*Ridleyandra chuana*, a new species of Gesneriaceae, is described and illustrated. It is endemic in Peninsular Malaysia and known from two small and restricted populations in montane forest. Its conservation status is assessed as vulnerable.

## Introduction

This species was first encountered in 1932 at Fraser’s Hill, Pahang. However, it was only in 1999 when another population was discovered by L.S.L. Chua on Gunung Ulu Kali, Pahang, that sufficient material was available for its description. Since then both these localities have been revisited and the Gunung Ulu Kali population is now the focus of conservation. This species is unusual in *Ridleyandra* in occupying a very narrow niche, which probably contributes to its rarity and small population size. This is clearly seen at the Fraser’s Hill locality where a small population of less than 30 plants is confined to a small area where moss-covered granite rock just emerges above the soil surface but is absent from the surrounded area of soil where *Codonoboea curtisii* (Ridl.) C.L.Lim (Gesneriaceae) is plentiful.

It is a distinctive species among *Ridleyandra* species in its mammillate leaf surface with hairs on raised conical bases. Indeed, when sterile with its dark green leaves it more resembles *Codonoboea crinita* (Jack) C.L.Lim than *Ridleyandra atrocyanea* (Ridl.) A.Weber,the only other *Ridleyandra* species with a mammillate leaf surface. Two species, *Ridleyandra kelantanensis* Kiew and *Ridleyandra longisepala* (Ridl.) A.Weber, have similar white corollas with purple lines and toothed leaves. In fact Weber (1998) listed Corner’s Fraser’s Hill specimens under *Ridleyandra longisepala* but it is different from this species that has bracts immediately below the sepals, longer petioles and sepals. It more resembles *Ridleyandra kelantanensis* in their shorter petioles and sepals.

## Taxonomy

### Ridleyandra chuana

Kiew sp. nov.

urn:lsid:ipni.org:names:77129808-1

http://species-id.net/wiki/Ridleyandra_chuana

Figure 1

#### Diagnosis.

In its leaf surface with mammillate hairs and its narrowly lanceolate bracts, *Ridleyandra chuana* resembles *Ridleyandra atrocyanea* (Ridl.) A.Weber but it differs in its shorter peduncles 6–8.5 cm long (not 8–12 cm), pedicels 5–7 mm long (not 10 mm) and its shorter 3–5 cm long white corolla with purple lines (not purple-black and 5–7 cm long). In flower colour and pattern it is similar to *Ridleyandra kelantanensis* Kiew but differs not only in its mammillate leaf surface but also in its longer petioles (2.3–3 cm long not 1–1.75 cm in *Ridleyandra kelantanensis*), smaller teeth on the leaf margin (2.5–3 × 1.5–3 mm not 4–5 × 3.5 mm), larger bracts (4–6 mm long not 2–3 mm), and corollas with a much narrower lip ca 13 mm wide (not 24 mm wide).

#### Type.

Peninsular Malaysia. Pahang: Gunung Ulu Kali, 27 Jan 1999 (fl.) LSL Chua FRI 40758 (holotype: KEP!; isotype: KEP!).

#### Description.

Perennial herb. **Stem** woody, usually unbranched, rarely 2-branched, erect, 12–19 cm tall, 3–5 mm diam., upper part of stem, petiole, leaf margin, lower surface of veins, peduncle and pedicel and sepals hispid; hairs reddish brown, dense, unbranched, multiseriate, ca. 2 mm long and on the margin and veins 3–4 mm long. **Leaves** in unequal pairs clustered in a rosette at the top of the stem, lowermost to 7 mm apart, deep green above, whitish green beneath; subsessile or lower leaves with petiole 2.3–3 cm long, ca. 6 mm diameter; lamina oblanceolate, 11–12.5(–14.5) × 2.7–4.5 cm, narrowed to base, margin serrate, teeth tip rounded, 2–3 × 1.5–3 mm, towards the base teeth divided almost to midrib, 5–9 × 2.5–4.5 mm, apex acute to acuminate, above hairs dense, surface mammillate with hairs raised on narrow cones, minutely punctate beneath; midrib impressed above, prominent beneath, lateral veins 17–18 pairs. **Inflorescence** 1-flowered, rarely 2-flowered then flowers open in succession, peduncle 6–8.5 cm long, dark maroon-purple, slightly curved upward; bract pair pale green, positioned 5–10 mm below the calyx, narrowly lanceolate, 4–6 × 0.75–2 mm, pedicel dark maroon-purple, 5–7 mm long. **Flowers** with sepals divided to base, dull purple or pale green with a red midrib, lanceolate, 4–7.5 × 1.5–2.5 mm, hispid; corolla funnel-shaped, 3–4 cm to tip of lower lip, tube 2–3 cm long, ca. 2 mm diam. at base dilating to 10 mm at the mouth, outside minutely pubescent, white at the base becoming tinged purple at the tube dilates, inside white with mauve or purple lines with 3 lines extending into each of the three lobes where they spread and coalesce leaving a white margin around each lobe, lobes projecting ca 10 mm beyond the tube, lateral lobes ca. 4 × 5 mm and the centre lobe ca. 5 × 6 mm; stamens 4 in 2 pairs, filaments white, lower pair ca. 23 mm long, upper pair ca. 26 mm long, anthers creamy white, ca 1 mm long, joined in pairs, staminode ca. 3 mm long; nectary annular, ca. 1 mm high; ovary ca. 3 cm long, pale mauve, stigma white, broadly spathulate, ca. 2 × 1.5 mm long, apex emarginated. **Capsules** glossy, deep purple, slightly curved upward, glabrous, 5–6.5 cm long, 2.5–4 mm diam., sepals persistent and clasping the base.

**Figure 1.**
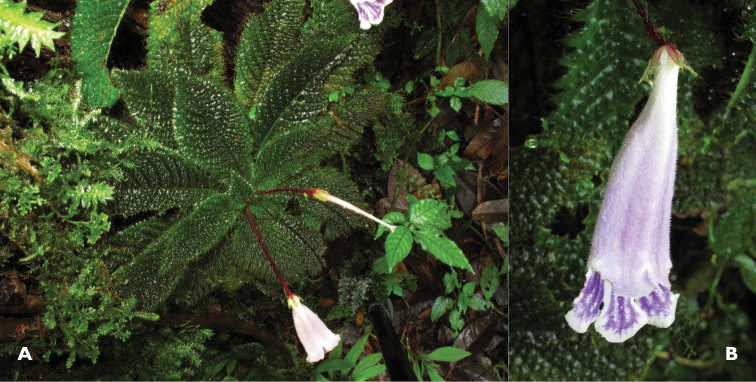
*Ridleyandra chuana* Kiew. **A** habit **B** flower.

#### Distribution.

**Map 1.**
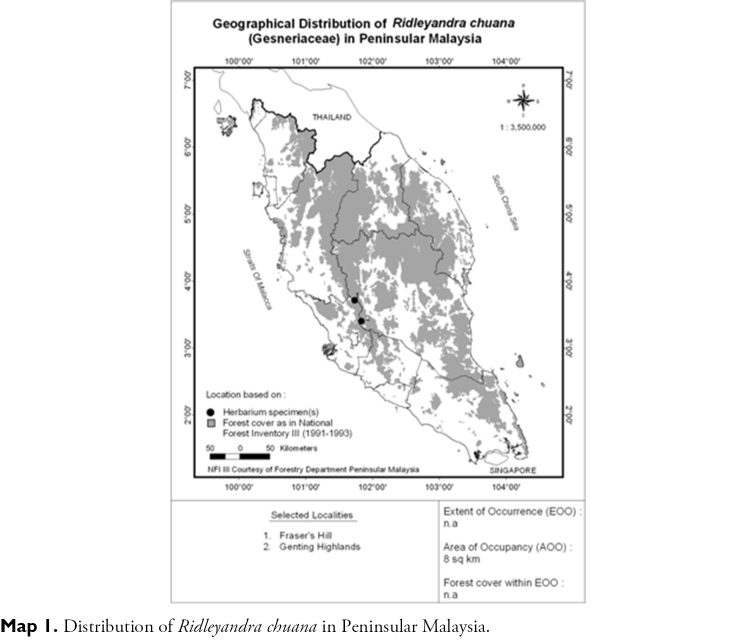
Distribution of *Ridleyandra chuana* in Peninsular Malaysia.

Endemic in Peninsular Malaysia, Pahang (Fraser’s Hill and Gunung Uli Kali) (Map 1).

#### Ecology.

On moss-covered granite rock embedded in soil or on low moss-covered granite boulders, in extremely damp, deeply shaded conditions on steep slopes in valleys. One population occurs in lower montane forest at 1250 m and the other in upper montane forest at–1570 m.

#### Etymology.

Named in honour of Dr Lillian Swee Lian Chua, botanist and conservationist, who first discovered this species on Gunung Kali while making an ecological inventory of the summit flora (Chua and Saw 2001).

#### Conservation status.

EN B2ab(ii,iii). Following the 2001 IUCN Red List Categories and Criteria, (IUCN 2001) this species is assessed as Endangered because it is known from two localities, one of which is threatened and only 130 known individuals. The population at Fraser’s Hill falls within a Totally Protected Area (Chua 2010) and consists of about 30 plants that grow in an undisturbed site away from tourist trails and is too remote to be affected by development. The other population consists of less than 100 plants at Gunung Ulu Kali, which is on private land in a hill resort that is severely threatened by road widening and associated landslips, by changes in microclimate due to edge effect as the forest becomes more and more fragmented and from and that is in danger of encroachment from future development. The chance of this latter population surviving is very slim. On the other hand, the rediscovery of the Fraser’s Hill population after a hundred years illustrates the resilience of species to survive if the habitat remains undisturbed.

#### Other specimens examined. Peninsular Malaysia.

Pahang: Gunung Ulu Kali–26 Nov 2007 LSL Chua & R Kiew FRI 46936 (KEP!); Fraser’s Hill– 4 Nov 1932 EJH Corner s.n. (SING!), 16 Aug 1937 EJH Corner s.n. (SING!), 18 Nov 2007 MY Chew et al. FRI 53772 (KEP!), 24 Feb 2008 R Kiew RK 5412 (KEP!).

#### Discussion.

Plants in both populations are identical in all characters except for sepal length (6.5–7.5 mm in the Ulu Kali population and ca. 4 mm in the Fraser’s Hill population).

In both the original collections (Chua FRI 40758 and Corner s.n. 1932) only a single flowering specimen was collected suggesting that this is not a free-flowering species. Monitoring over a longer period by JPC Tan suggests that there is a low level of flowering throughout the year with periodic bursts of more intense flowering. This same pattern is seen in some species of *Codonoboea*, such as *Codonoboea platypus* (C.B.Clarke) C.L.Lim (Kiew 2009b).

## Supplementary Material

XML Treatment for Ridleyandra chuana

## References

[B1] Chua LSL (2010) Species assessment and conservation in Peninsular Malaysia. In: Kiew R, Chung RCK, Saw LG, Soepadmo E, Boyce PC (Eds) Flora of Peninsular Malaysia 2,1: 47–54.

[B2] Chua LSL Saw LG (2001) A reassessment of the flora of Gunung Ulu Kali, Genting Highlands, Malaysia–preliminary findings and trends. Malayan Nature Journal 55: 65-76

[B3] IUCN (2001) IUCN Red List Categories and Criteria version 3.1. IUCN Species Survival Commission, IUCN, Gland, Switzerland and Cambridge, U.K.

[B4] Kiew R (2009a) Three new species of Gesneriaceae from Kelantan, Malaysia. Gardens Bulletin Singapore 61: 73-79

[B5] Kiew R (2009b) The natural history of Malaysian Gesneriaceae. Malayan Nature Journal 61: 257–265.

[B6] Kiew R Lim CL (2011) Names and new combinations for Peninsular Malaysian species of *Codonoboea* Ridl. (Gesneriaceae). Gardens Bulletin Singapore 62: 253-275

[B7] Ridley HN (1905) The Gesneriaceae of the Malay Peninsula. Journal Straits Branch Royal Asiatic Society. 44: 1-92

[B8] Weber A (1998 [‘1997’]) Revision of the genus *Ridleyandra* (Gesneriaceae). Beiträge zur Biologie der Pflanzen 70: 225−273

